# Gene expression profile and synovial microcirculation at early stages of collagen-induced arthritis

**DOI:** 10.1186/ar1754

**Published:** 2005-05-17

**Authors:** Philip Gierer, Saleh Ibrahim, Thomas Mittlmeier, Dirk Koczan, Steffen Moeller, Jürgen Landes, Georg Gradl, Brigitte Vollmar

**Affiliations:** 1 Department of Experimental Surgery, University of Rostock, Rostock, Germany; 2 Department of Trauma & Reconstructive Surgery, University of Rostock, Rostock, Germany; 3 Institute of Immunology, University of Rostock, Rostock, Germany; 4 Department of Surgery, Klinikum Innenstadt, Ludwig Maximilians University, Munich, Germany

## Abstract

A better understanding of the initial mechanisms that lead to arthritic disease could facilitate development of improved therapeutic strategies. We characterized the synovial microcirculation of knee joints in susceptible mouse strains undergoing intradermal immunization with bovine collagen II in complete Freund's adjuvant to induce arthritis (i.e. collagen-induced arthritis [CIA]). Susceptible DBA1/J and collagen II T-cell receptor transgenic mice were compared with CIA-resistant FVB/NJ mice. Before onset of clinical symptoms of arthritis, *in vivo *fluorescence microscopy of knee joints revealed marked leucocyte activation and interaction with the endothelial lining of synovial microvessels. This initial inflammatory cell response correlated with the gene expression profile at this disease stage. The majority of the 655 differentially expressed genes belonged to classes of genes that are involved in cell movement and structure, cell cycle and signal transduction, as well as transcription, protein synthesis and metabolism. However, 24 adhesion molecules and chemokine/cytokine genes were identified, some of which are known to contribute to arthritis (e.g. CD44 and neutrophil cytosolic factor 1) and some of which are novel in this respect (e.g. CC chemokine ligand-27 and IL-13 receptor α_1_). Online *in vivo *data on synovial tissue microcirculation, together with gene expression profiling, emphasize the potential role played by early inflammatory events in the development of arthritis.

## Introduction

Murine collagen-induced arthritis (CIA) is a chronic inflammatory disease that bears all the hallmarks of rheumatoid arthritis (RA), namely polyarthritis and synovitis with subsequent cartilage and bone erosions [1]. CIA is induced in susceptible strains of mice (e.g. DBA1/J) by immunization with bovine collagen type II in complete Freund's adjuvant (CFA). The development of CIA is thought to depend on T cells, and disease susceptibility is linked to the major histocompatibility region [2]. Activated lymphocytes migrate to the joint, where an inflammatory cascade involving T cells, macrophages, monocytes, B cells and activated synoviocytes is triggered. This cellular infiltration, together with production of a complex array of cytokines and other soluble mediators, contributes to synovial proliferation, pannus formation, cartilage destruction and subchondral bone erosion [3].

Because the inflammatory process within joint tissues represents a key feature of RA, an understanding of the mechanisms that induce and sustain this aspect of RA pathology would permit development of new and powerful therapeutic strategies. With direct online visualization, the technique of intravital fluorescence microscopy permits dissection of the complex cell inflammatory response, with differentiation between cellular subtypes and their distinct adhesion molecule dependent interactions within the microcirculation.

The approach of *in vivo *microscopy has successfully been applied in joints of mice with antigen-induced arthritis (AIA) [4]. AIA is established in mice by immunizing them with methylated bovine serum albumin (mBSA) in CFA with or without an arthritogenic infectious agent at days 0 and 7, followed by intra-articular injection of mBSA at day 21 [5]. Although AIA is an established animal model for the study of human RA [6], arthritis is more commonly induced using collagen, and this represents the primary animal model for RA in humans [7-9]. Therefore, we employed *in vivo *microcirculatory analysis of knee joints in mice with CIA, using different strains that are known to acquire CIA, such as DBA1/J mice and T-cell receptor (TCR) transgenic mice that carry the rearranged Vα11.1 and Vβ8.2 chain genes isolated from a type II collagen-specific T-cell hybridoma (DBA-CII-TCR Tg) [10]. FVB/NJ mice were used as controls, because these mice have been reported to be resistant to arthritis induction, probably because of a genomic deletion of TCR Vβ gene segments [11].

Because we were particularly interested in the disease initiation stage, the synovial microcirculation was assessed before the onset of clinical symptoms of arthritis. We further characterized the global gene expression profile at this early stage in the disease in order to define the initial molecular mechanisms and to determine the onset of joint inflammation.

## Materials and methods

### Animal model

The experimental protocol was approved by the local animal rights protection authorities (LVL M-V/TSD/7221.3-1.1-037/04) and followed the National Institutes of Health guidelines for the care and use of laboratory animals. DBA1/J and FVB/NJ mice were obtained from the Jackson Laboratory (Bar Harbor, ME, USA). Collagen II specific TCR transgenic mice were a kind gift from Professor Ladiges, University of Washington, USA [10]. All mice were kept under standard conditions at the animal care facility of the University of Rostock.

Mice aged 8 weeks (*n *= 5–10 per strain and group) were immunized intradermally at the base of the tail with 125 μg bovine collagen II (Chondrex, Redmond, WA, USA) emulsified in CFA (DIFCO, Detroit, MI, USA) or with equivalent volumes of CFA only. Six weeks after immunization and before clinical signs of arthritis manifested, animals were anaesthetized with ketamine (90 mg/kg body weight) and xylacin (6 mg/kg) and placed on a heating pad to maintain their body temperature at 37°C. A catheter was placed in the left jugular vein for application of fluorescent dyes.

For *in vivo *multifluorescence microscopy of synovial microcirculation, we used the knee joint model initially described by Veihelmann and coworkers [4]. Briefly, the skin was incised distal to the patella tendon. After removal of the overlying soft tissues, the patella tendon was transversally cut and the proximal and distal part carefully mobilized. After exposure, the 'Hoffa's fatty body' was superfused with 37°C warm physiological saline solution to prevent the tissues from drying and finally covered with a glass slide. Following a 15-min stabilization period after surgical preparation, *in vivo *microscopy of the synovial tissue was performed. At the end of the experiments, animals were killed by exsanguination. The complete knee joint was excised and harvested for subsequent histology. Paws were used for gene expression profile analysis.

### Clinical evaluation of arthritis

As described by Nanakumar and coworkers [12], scoring of animals was done blindly using a scoring system based on the number of inflamed joints in each paw, inflammation being defined by swelling and redness. In this scoring system each inflamed toe or knuckle is attributed 1 point, whereas an inflamed wrist or ankle is attributed 5 points, resulting in a score of 0 to 15 (five toes + five knuckles + one wrist/ankle) for each paw and 0–60 points for each mouse [12].

### *In vivo *fluorescence microscopy

After intravenous injection of FITC-labelled dextran (15 mg/kg body weight; Sigma, Deisenhofen, Germany) and rhodamine 6G (0.15 mg/kg body weight; Sigma), *in vivo *microscopy was performed using a Zeiss microscope (Axiotech vario 100HD; Carl Zeiss, Oberkochen, Germany) equipped with a 100 W mercury lamp and filter sets for blue (excitation 465–495 nm, emission >505 nm) and green (excitation 510–560 nm, emission >575 nm) epi-illumination. Using water-immersion objectives (×20 W/numerical aperture 0.5 and ×40 W/numerical aperture 0.8; Carl Zeiss), final magnifications of 306× and 630× were achieved. Images were recorded by means of a charge-coupled device video camera (FK 6990-IQ-S; Pieper, Schwerte, Germany) and transferred to a S-VHS video system for subsequent offline analysis.

### Microcirculatory analysis

For quantitative offline analysis a computer-assisted microcirculation image analysis system was used (CapImage v7.4; Zeintl, Heidelberg, Germany). Functional capillary density was defined as the total length of red blood cell perfused capillaries per observation area, and is given in cm/cm^2^. For assessment of leucocyte–endothelial cell interaction in postcapillary venules, flow behaviour of leucocytes was analyzed with respect to free floating, rolling and adherent leucocytes. Rolling leucocytes were defined as those cells moving along the vessel wall at a velocity less than 40% that of leucocytes at the centre line, and are expressed as a percentage of the total leucocyte flux. Venular leucocyte adherence was defined as the number of leucocytes not moving or detaching from the endothelial lining of the venular vessel wall during an observation period of 20 s. Assuming cylindrical microvessel geometry, leucocyte adherence was expressed as nonmoving cells per endothelial surface (*n*/mm^2^), calculated from the diameter and length of the vessel segment analyzed. In postcapillary venules, centre line red blood cell velocity (V_RBC_) was determined using the line shift method (CapImage; Zeintl, Heidelberg, Germany). The wall shear rate was calculated based on the Newtonian definition: y = 8 × V_mean_/D, where V_mean _is the mean velocity (V_RBC_/1.6) and D is diameter of the individual microvessel.

### Laboratory analysis

Arterial blood samples were drawn for analysis of blood cell counts using a Coulter Counter (AcTdiff; Coulter, Hamburg, Germany).

### Sample preparation for high-density oligonucleotide microarray hybridization

Paws of DBA/1J mice immunized with CFA or CFA/collagen II and unimmunized mice were dissected and snap frozen in liquid nitrogen, and total RNA was extracted using a commercially available system (Qiagen, Hilden, Germany). RNA probes were labelled in accordance with the manufacturer's instructions (Affymetrix, Santa Clara, CA, USA). Analysis of gene expression was conducted using the U430A array (Affymetrix), which has a capacity of about 20,000 genes. Samples from individual mice were hybridized onto individual arrays. Hybridization and washing of gene chips were done in accordance with the manufacturer's instructions and were as described previously [13]. Microarrays were analyzed by laser scanning and the expression levels were calculated using commercially available software provided by Affymetrix [13]. The files were then analyzed using the affylmGUI package of the Bioconductor software suite (Affymetrix) [14,15]. The expression was determined using the robust multichip average method [16]. A linear model of the expression data for Limma was created within affylmGUI, for which the six arrays of mice immunized with CFA/collagen II, the three arrays for mice administered CFA only, and the two arrays for control mice were separated into three groups. Contrasts were calculated for each group against the other two.

**Figure 1:**
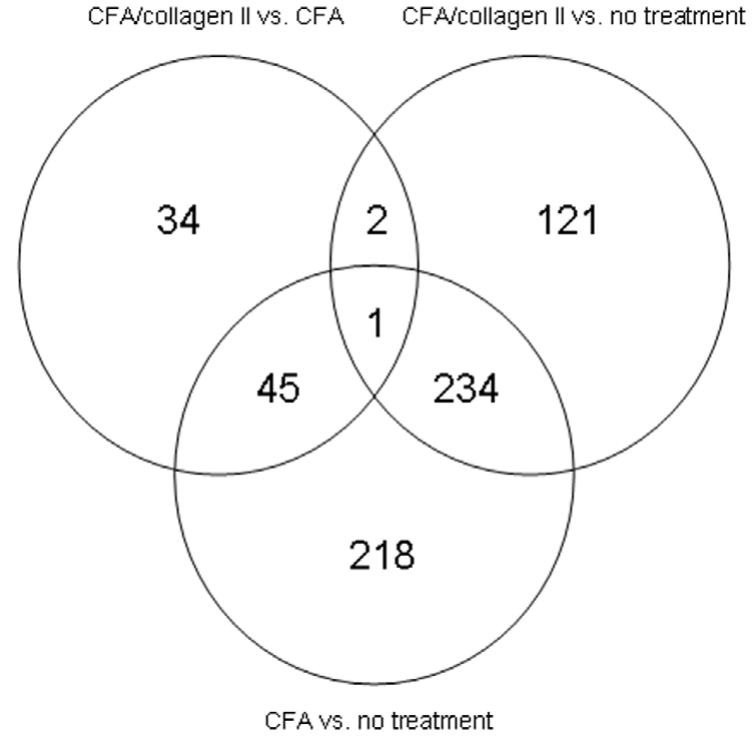
Summary of differentially expressed genes at the early stage of arthritis. Of the approximately 22,000 genes on the Affymetrix chip MOE430a, 655 genes were significantly differentially expressed (minimum 1.5-fold; *P *< 0.001) in a particular comparison: complete Freund's adjuvant (CFA)/collagen II versus CFA, CFA/collagen II versus no treatment, or CFA versus no treatment. Genes differentially expressed in more than one comparison are shown.

The expression data are available in Additional files 1, 2, 3. Genes considered differentially expressed were selected on the basis of *P *value (<0.001) and a 1.5-fold change in intensity (abs [M value] = log2 [1.5]). These genes are presented in Additional file 4. Gene Ontology (GO) terms were assigned to the selected genes (Fig. 1 and Additional file 4) via the Bioconductor GO package 1.6.8 and the chip annotation package MOE430a of the same version [17]. The Bioconductor GO package provides lists of reachable subterms for each GO term. We used this function to filter genes associated with adhesion, specifically those assigned to 'adhesion offspring' for any term in the following list: GO:0005125 (cytokine activity), GO:0006955 (immune response), GO:0050776 (regulation of immune response), GO:0004895 (cell adhesion receptor activity), GO:0007155 (cell adhesion), GO:0016337 (cell–cell adhesion), GO:0030155 (regulation of cell adhesion), GO:0050839 (cell adhesion molecule binding), GO:0030155 (regulation of cell adhesion), GO:0019955 (cytokine binding), GO:0005912 (adherens junction), GO:0005925 (focal adhesion), GO:0050900 (immune cell migration), GO:0030595 (immune cell chemotaxis), GO:0006954 (inflammatory response) and GO:0006935 (chemotaxis).

### Histology

At the end of each experiment, knee joints were fixed in 4% phosphate-buffered formalin for 2–3 days, decalcified in EDTA for 6 weeks, and then embedded in paraffin. From the paraffin-embedded tissue blocks, 4 μm sections were cut and stained with haematoxylin–eosin for histological analysis. For semiquantitative analysis, the score described by Brackertz and coworkers [6] was used (0 = normal knee joint; 1 = occasional mononuclear cells in normal synovium; 2 = perivascular leucocyte infiltration, two or more synovial cell layers; 3 = dense infiltration of leucocytes, synovial hyperplasia; 4 = synovitis, pannus formation and cartilage erosions). The analysis was done by a blinded and independent observer.

### Statistical analysis of microcirculatory data

Results are presented as mean ± standard error of the mean. After proving the assumption of normality, comparisons between the experimental groups were performed by one-way analysis of variance, followed by the appropriate *post hoc *multiple comparison procedure, including Bonferroni correction (SigmaStat; Jandel, San Rafael, CA, USA). *P *< 0.05 was considered statistically significant.

## Results

### Gene expression profile in joints at onset of arthritis

To define the gene expression profile at early stages of CIA, we used the murine Affymetrix oligonucleotide microarray MOE430a, with more than 20,000 gene specificities, to compare the three groups of mice (i.e. CFA/collagen II immunized, CFA immunized and unimmunized). As shown in Additional files 1, 2, 3, 4 and Fig. 1, 655 genes were differentially expressed between groups and taking a *P *< 0.001 as the threshold for significance.

Interestingly, CFA alone induced the greatest number of differentially expressed genes (i.e. 498). A total of 375 genes overlapped between different groups, and 280 were unique to a certain group of mice (Additional file 4). When grouped according to their probable function (i.e. GO terms), the majority of the differentially expressed genes fell into classes of genes involved in cell movement and structure, cell cycle and signal transduction, as well as transcription, protein synthesis and metabolism. One prominent group was that of adhesion molecules and chemokine/cytokine-related genes. Twenty-four genes belonging to this group were identified and are summarized in Table 1 (Additional file 4). Some genes are well known for their contribution to cell activation, cell–cell communication and chemotaxis, such as CD44, CD36, IL-1 receptor antagonist and neutrophil cytosolic factor (NCF)-1, as well as CC and CXC chemokines (Table 1, Additional file 4).

### Systemic parameters

The animals from the CFA/collagen II immunized and the CFA immunized groups did not differ with respect to haemoglobin and haematocrit (Table 2). Moreover, there were no differences in blood cell counts between the two groups (Table 2).

### Microvascular perfusion in synovial tissue

**Figure 2:**
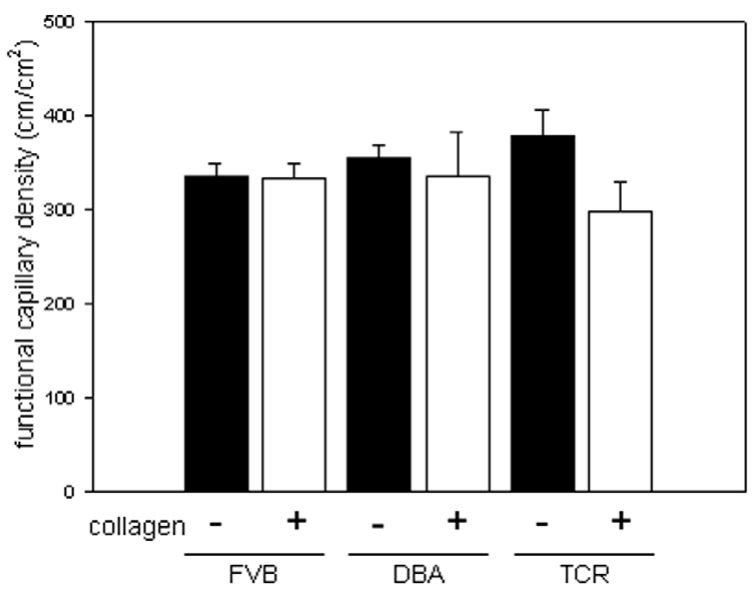
Functional capillary density. Shown is the functional capillary density of the synovium in complete Freund's adjuvant (CFA)/collagen II exposed FVB/NJ, DBA1/J and T-cell receptor (TCR) transgenic mice (collagen +) in comparison with CFA treated controls (collagen -). Intravital fluorescence microscopy of the knee joints was performed at 6 weeks after collagen exposure for induction of arthritis. Values are expressed as means ± standard error (*n *= 5–10 animals/group).

Functional capillary density did not differ significantly between the experimental groups (Fig. 2), although there was a tendency toward lower values in the DBA mice, and in particular the TCR transgenic mice, after CFA/collagen II exposure (Fig. 2). Capillary diameters increased and V_RBC _decreased in all animals exposed to CFA/collagen II exposure compared with those subjected to CFA control treatment (Table 3). Wall shear rates were found to be reduced in the CFA/collagen II treated mice in comparison with those treated with CFA (Table 3).

### Inflammatory cell response in synovial tissue

**Figure 3:**
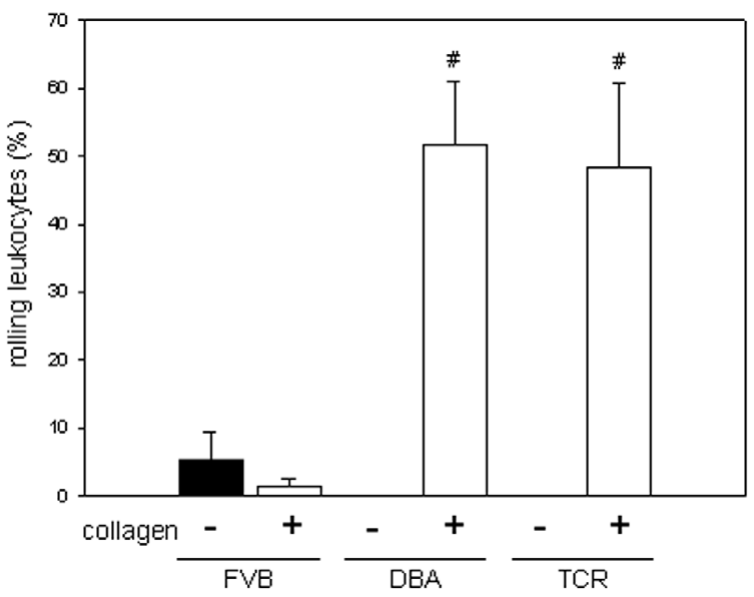
Leucocytes rolling along the endothelium of postcapillary synovial venules. Shown are the proportions of leucocytes rolling along the endothelium of postcapillary synovial venules (as % of all passing leucocytes) in complete Freund's adjuvant (CFA)/collagen II exposed FVB/NJ, DBA1/J and T-cell receptor (TCR) transgenic mice (collagen +) in comparison with CFA treated controls (collagen -). Intravital fluorescence microscopy of the knee joints was performed at 6 weeks after collagen exposure for induction of arthritis. Values are expressed as means ± standard error (*n *= 5–10 animals/group); analysis of variance, unpaired *post hoc *comparison test: ^#^*P *< 0.05 versus corresponding CFA-treated control animals (collagen -).

**Figure 4:**
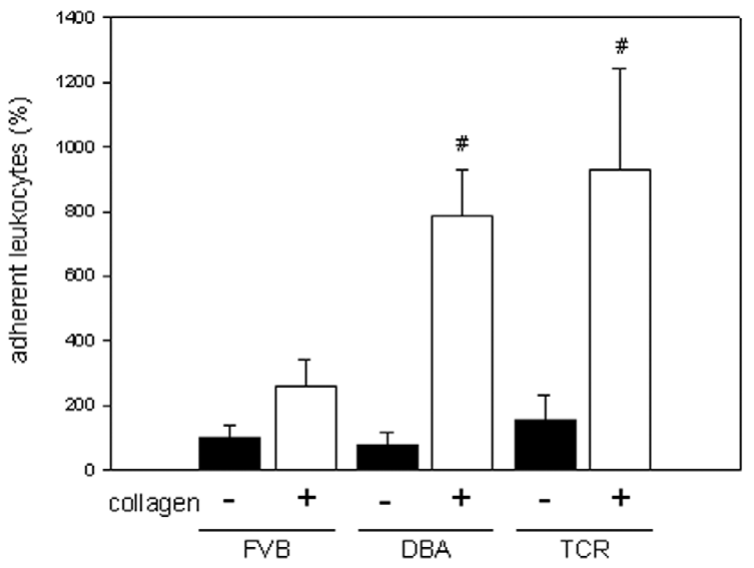
Leucocytes adherent to the endothelium of postcapillary synovial venules. Shown are the numbers of leucocytes adherent to the endothelium of postcapillary synovial venules (cells/mm^2 ^endothelial surface) in complete Freund's adjuvant (CFA)/collagen II exposed FVB/NJ, DBA1/J and T-cell receptor (TCR) transgenic mice (collagen +) in comparison with CFA treated controls (collagen -). Intravital fluorescence microscopy of the knee joints was performed at 6 weeks after collagen exposure for induction of arthritis. Values are expressed as mean ± standard error (*n *= 5–10 animals/group); analysis of variance, unpaired *post hoc *comparison test: ^#^*P *< 0.05 versus corresponding CFA-treated control animals (collagen -).

Although the animals exhibited no clinical symptoms of arthritic disease, synovial tissue was characterized by an inflammatory cell response with significant (*P *< 0.05) increases in leucocytes, both rolling along and firmly attaching to the venular endothelium, in DBA and TCR transgenic mice (Figs 3 and 4). In contrast, CIA-resistant FVB animals did not respond with enhanced leucocyte–endothelial cell interaction on CFA/collagen II exposure, with findings equivalent to those in CFA treated control animals (Figs 3 and 4). Concomitant with the lack of clinical signs, the score in haematoxylin and eosin stained knee joints was found to be less than 1 in all animals, irrespective of genotype and treatment (data not shown).

## Discussion

In the present study we found that susceptible mice that were exposed to CFA/collagen II for induction of arthritis exhibited marked signs of inflammation within the microcirculation of the knee joint, although animals were still free from clinical symptoms. Collagen II treated TCR transgenic and DBA/1J mice did not differ in terms of the extent of inflammation, which exceeded that in resistant FVB animals markedly. The inflammatory cell response, as indicated by the enhanced activation and interaction of leucocytes with the microvascular endothelium, was mirrored by the expression of genes that contribute to cell activation, cell–cell communication and chemotaxis.

Despite the considerable work done to elucidate disease pathways, several aspects of RA remain poorly defined. A rigorous understanding of the initial mechanisms involved in the pathogenesis of RA would permit the development of strategies to impede the manifestation of the disease. In numerous organ pathologies, the activation of circulating leucocytes, and their interaction with the endothelial lining followed by subsequent transendothelial migration and infiltration into tissue represent the first and determining step in a complex sequence of processes that mediate tissue injury [18-20]. In contrast to our previous study addressing the expression profile of joints in CIA mice at the peak of the disease [13], we intentionally focused on an early stage in the disease, in which the animals were free of clinical symptoms. Although arthritic disease with establishment of pannus tissue is dominated by genes that are involved in cell division and proliferation, rather than immunologically relevant genes [13], early disease appears to be characterized by distinct upregulation of a group of chemotactic and adhesion molecules, such as CD44 and IL-13 receptor α_1_, as well as CC chemokine ligand (CCL)-24 and CCL-27, which presumably are responsible for cell attraction within the joint microcirculation. Many of those molecules were induced by both CFA and CFA/collagen II treatment, which is unsurprising because CFA is essential for induction of CIA.

Interestingly, the only upregulated adhesion molecule in the comparison between mice treated with CFA/collagen II and those treated with CFA alone was CD44, supporting a role for CD44 in this early stage of arthritis. Indeed, there is considerable published evidence for CD44 involvement in arthritis, although its exact role remains controversial [21,22].

In accord with the importance of adhesion molecules in development of arthritis, frozen section binding assays in rheumatoid synovitis demonstrated that, apart from E-selectin and counter receptors for β_1_/β_2 _integrins, P-selectin is the predominant adhesion molecule, mediating monocyte binding to inflamed synovial venules [23]. Similarly, increased cellular infiltration and increased expression of E-selectin, intercellular adhesion molecule-1, vascular cell adhesion molecule-1, platelet/endothelial cell adhesion molecule-1, very late appearing antigen-4, and Mac-1 were found in immunohistochemistry of synovial tissue from patients with RA [24]. Veihelmann and coworkers [25] demonstrated high numbers of adherent leucocytes upon clinical manifestation of AIA in mice, regardless of phase (acute, intermediate, or chronic) of disease. In accordance with and extending the findings of the latter study, we now show that leucocyte adhesion is apparent even if clinical symptoms are still absent, underscoring leucocyte–endothelial interaction as an integral part not only of the perpetuation and propagation of disease but also of its initiation.

Apart from adhesion molecules, a few chemokines and inflammatory mediators were found among the genes predominantly expressed in CIA mice in the present study. This is in accordance with the common knowledge that the key mechanisms underlying synovitis include inflammatory cell activation and adhesion, as well as production of mediators such as cytokines, chemokines and growth factors [26,27]. In particular, tumour necrosis factor (TNF)-α and IL-1 regulate nuclear factor-κB inducible genes that control – apart from other factors – cell adhesion molecules, proinflammatory mediators and immunomodulatory molecules. These properties established a rationale for anticytokine therapeutics and their evaluation in an extensive series of clinical trials [28]. Anti-TNF-α therapy has been shown to reduce expression of adhesion molecules and to decrease cellularity of rheumatoid synovial tissue [29,30], supporting the hypothesis that the anti-inflammatory effect is due to a downregulation of cytokine-inducible vascular adhesion molecules with a consequent reduction in cell traffic into joints.

A few molecules belonging to the category of chemokines and inflammatory mediators were differentially expressed and deserve further investigation. These are NCF-1, IL-13 receptor α_1 _and CCL-27. NCF-1 is a member of the NADPH (nicotinamide adenine dinucleotide phosphate, reduced form) oxidase complex, which was recently identified as a susceptibility gene for pristine-induced arthritis. However, its exact role in disease remains unclear [31]. The chemokine ligand CCL-27 was recently shown to bind the P-selectin glycoprotein ligand 1 – a molecule that plays a role in homing of T lymphocytes [32]. The role played by the cytokine receptor IL-13 receptor α_1 _in arthritis has not been established, but its ligand, IL-13, has been described as a cytokine with anti-inflammatory properties in arthritis and was the target of experimental gene therapy experiments [33].

Of interest, mice from the two strains studied did not differ with respect to functional capillary density, averaging about 320 cm/cm^2^. Corresponding values were found in Balb/c mice during acute and intermediate phases of AIA [21] but were attributed to inflammation-associated angiogenesis, because control animals had values well below 250 [4,21]. If it were angiogenesis driven, this would not account for the high functional capillary density in CFA treated control animals of the present study. Thus, it is more likely that differences in functional capillary density are simply due to the fact that different strains were used.

## Conclusion

Our data suggest that upregulation of proinflammatory mediators and molecules facilitate leucocyte adhesion to the endothelium and migration into tissue, thereby representing an essential and primary step in the development of arthritis. Although studies of early RA are few, because there is an inherent delay before patients receive expert care, it has been recognized that early intervention improves outcome. Thus, the early innate immune response should be an ongoing focus of future research to determine whether leucocyte activation predicts severity of disease and is the earliest change to occur in rheumatoid synovium.

## Abbreviations

AIA = antigen-induced arthritis; CCL = CC chemokine ligand; CFA = complete Freund's adjuvant; CIA = collagen-induced arthritis; GO = Gene Ontology; IL = interleukin; mBSA = methylated bovine serum albumin; NCF = neutrophil cytosolic factor; RA = rheumatoid arthritis; TCR = T-cell receptor; TNF = tumour necrosis factor; V_RBC _= centre line red blood cell velocity.

## Competing interests

The author(s) declare that they have no competing interests.

## Authors' contributions

PG, JL and GG performed the animal experiments and intravital fluorescence microscopic analysis. SI, DK and SM performed gene expression profiling experiments with bioinformatic analysis. SI, TM and BV conceived the study, and participated in its design and coordination. SI and BV drafted the manuscript. All authors read and approved the final manuscript.

## Supplementary Material

Additional File 1 Excel file showing the output of the topTable function of the GNU R Limma package, as provided by the R affylmGUI package: complete Freund's adjuvant (CFA)/collagen II versus CFA. The columns first describe the genes using an internal identification number (ID), the Affymetrix probe set ID, the gene's symbol and a small description or full name. The statistics for the genes are summarized in the columns M (logarithmic fold change; the difference in logarithm of expression for each group), A (logarithmic mean expression), t (moderated t statistic), *P *value (nominal *P *value) and B (log odds that the gene is differentially expressed). With B positive, the gene is more likely to be differentially expressed than not. At 0 it is uncertain. Click here for file

Additional File 2 Excel file showing the output of the topTable function of the GNU R limma package as provided by the R affylmGUI package: complete Freund's adjuvant (CFA)/collagen II versus no treatment. The columns first describe the genes using an internal identification number (ID), the Affymetrix probe set ID, the gene's symbol and a small description or full name. The statistics for the genes are summarized in the columns M (logarithmic fold change; the difference in logarithm of expression for each group), A (logarithmic mean expression), t (moderated t statistic), *P *value (nominal *P *value) and B (log odds that the gene is differentially expressed). With B positive, the gene is more likely to be differentially expressed than not. At 0 it is uncertain. Click here for file

Additional File 3 Excel file showing the output of the topTable function of the GNU R limma package as provided by the R affylmGUI package: complete Freund's adjuvant (CFA) vs. no treatment. The columns first describe the genes using an internal identification number (ID), the Affymetrix probe set ID, the gene's symbol and a small description or full name. The statistics for the genes are summarized in the columns M (logarithmic fold change; the difference in logarithm of expression for each group), A (logarithmic mean expression), t (moderated t statistic), *P *value (nominal *P *value) and B (log odds that the gene is differentially expressed). With B positive, the gene is more likely to be differentially expressed than not. At 0 it is uncertain. Click here for file

Additional File 4 Excel file summarizing genes differentially expressed in collagen-induced arthritis (CIA) joints at early stages in the disease. The first column gives the probe set identification number (ID) of the Affymetrix chip Moe430a. Columns 2–4 list whether the gene is significantly upregulated (Up) or downregulated (Dn; minimum 1.5-fold; *P *< 0.001) in a particular comparison, ordered complete Freund's adjuvant (CFA)/collagen II versus CFA, CFA/collagen II versus no treatment, and CFA versus no treatment. Columns 5 and 6 show the gene symbol and its name. For further details, see Materials and method. Click here for file

## Figures and Tables

**Table 1:** Summary of adhesion molecules and chemokines/cytokines differentially expressed in CIA joints at early stages of disease and their GO terms.

Affymetrix ID	Change	Gene ID	Description	Go Term/Function
Solely changed in comparison CFA/collagen II versus CFA				
1423760_at	Up	*Cd44*	CD44 antigen	GO:0007155 cell adhesion
Solely changed in comparison CFA/collagen II versus no treatment				
1422103_a_at	Dn	*Stat5b*	Signal transducer and activator of transcription 5B	GO:0030155 cell adhesion
1423166_at -	Dn	*Cd36*	CD36 antigen	GO:0007155 cell adhesion
1427165_at -	Up	*Il13ra1*	IL-13 receptor alpha 1	GO:0004907 IL activity
1434044_at -	Dn	*Repin1*	Replication initiator 1	GO:0006954 inflammatory response
1452483_a_at	Up	*Cd44*	CD44 antigen	GO:0007155 cell adhesion
1452514_a_at	Dn	*Kit*	Kit oncogene	GO:0006935 chemotaxis
Solely changed in comparison CFA versus no treatment				
1416156_at	Dn	*Vcl*	Vinculin	GO:0005912 adherens junction
1417705_at	Dn	*Otub1*	OTU domain, ubiquitin aldehyde binding 1	GO:0006955 immune response
1422873_at	Dn	*Prg2*	Proteoglycan 2, bone marrow	GO:0006955 immune response
1430375_a_at	Up	*Ccl27*	Chemokine (CC motif) ligand 27	GO:0008009 chemokine activity
1437807_x_at	Dn	*Catna1*	Catenin α_1_	GO:0005912 adherens junction
1452020_a_at	Up	*Siva*	CD27 binding protein	GO:0005175 CD27 receptor binding
1455158_at	Dn	*Itga3*	Integrin α_3_	GO:0007155 cell adhesion
Changed in comparisons CFA/collagen II vs. CFA and CFA vs. no treatment				
1450488_at	Dn-Up	*Ccl24*	Chemokine (CC motif) ligand 24	GO:0008009 chemokine activity
Changed in comparisons CFA/collagen II vs. no treatment and CFA vs. no treatment				
1419329_at	Dn Dn	*Sh3d4*	SH3 domain protein 4	GO:0007155 cell adhesion
1420465_s_at	Up Up	Mup1	Major urinary protein 1	GO:0016068 type I hypersensitivity
1420553_x_at	Dn Dn	*Serpina1a*	Serine proteinase inhibitor, clade A, member 1a	GO:0006953 acute-phase response
1423017_a_at	Up Up	*Il1rn*	IL-1 receptor antagonist	GO:0006955 immune response
1423734_at	Up Up	*Rac1*	RAS-related C3 botulinum substrate 1	GO:0007155 cell adhesion
				GO:0006954 inflammatory response
1423885_at	Dn Dn	*Lamc1*	Laminin γ_1_	GO:0007155 cell adhesion
1427164_at	Up Up	*Il13ra1*	IL-13 receptor α_1_	GO:0004907 IL activity
1435148_at	Dn Dn	*Atp1b2*	ATPase, Na^+^/K^+ ^transporting, β_2 _polypeptide	GO:0007155 cell adhesion
1448303_at	Dn Dn	*Gpnmb*	Glycoprotein nmb	GO:0007155 cell adhesion
1451767_at	Up Up	*Ncf1*	Neutrophil cytosolic factor 1	GO:0006954 inflammatory response

Shown are differentially expressed genes associated with adhesion or inflammation with respect to Gene Ontology (GO) terms. The genes are a subset of those shown in the Venn diagram (Fig. 1). The first column gives the probe set identification number (ID) of the Affymetrix chip Moe430a. The second column lists whether the gene is significantly upregulated (Up) or downregulated (Dn; minimum 1.5-fold) in a particular comparison, ordered complete Freund's adjuvant (CFA)/collagen II versus CFA, CFA/collagen II versus no treatment, and CFA versus no treatment. Columns three to five show the gene symbol, its name and GO terms. For further details, see Materials and method. IL, interleukin.

**Table 2:** Haemoglobin, haematocrit and blood cell counts following CFA or CFA/collagen II exposure

	CFA			CFA/collagen II		
	
	FVB	DBA	TCR	FVB	DBA	TCR
Haemoglobin (mmol/l)	6.5 ± 0.4	7.1 ± 0.4	7.1 ± 0.4	7.1 ± 0.2	7.7 ± 0.3	7.9 ± 0.1
Haematocrit (%)	36 ± 2	43 ± 3	41 ± 3	40 ± 1	47 ± 2	46 ± 1
Thrombocytes (10^9^/l)	851 ± 96	447 ± 73	551 ± 124	984 ± 210	729 ± 124	743 ± 111
Leucocytes (10^9^/l)	3.8 ± 0.5	4.4 ± 0.9	2.1 ± 0.8	1.8 ± 0.5	4.9 ± 1.0	3.7 ± 0.7
Lymphocytes (%)	76 ± 5	80 ± 3	70 ± 3	65 ± 2	65 ± 4	65 ± 5
Mixed population (%)	14 ± 1	11 ± 2	26 ± 4	25 ± 3	32 ± 5	30 ± 4
Neutrophils (%)	6 ± 4	4 ± 1	5 ± 1	10 ± 3	3 ± 1	5 ± 2

Values are expressed as mean ± standard error. Blood samples were drawn at 6 weeks after complete Freund's adjuvant (CFA)- and CFA/collagen II exposure. TCR, T-cell receptor.

**Table 3:** Capillary diameter, venular red blood cell velocity and wall shear rates in synovial tissue following CFA or CFA/collagen II exposure

	CFA			CFA/collagen II		
	
	FVB	DBA	TCR	FVB	DBA	TCR
Capillary diameter (μm)	4.3 ± 0.1	4.5 ± 0.2	4.4 ± 0.1	4.5 ± 0.1	4.7 ± 0.1	4.6 ± 0.1
V_RBC _(μm/s)	840 ± 143	1085 ± 350	1760 ± 412	620 ± 278	835 ± 251	487 ± 122*
Wall shear rate (s^-1^)	0.20 ± 0.03	0.21 ± 0.05	0.40 ± 0.09	0.12 ± 0.03	0.18 ± 0.05	0.10 ± 0.02

Values are expressed as means ± standard error. *P < 0.05 versus corresponding complete Freund's adjuvant (CFA) treated control animals. TCR, T-cell receptor; V_RBC_, venular red blood cell velocity.
